# Purkinje cell vulnerability induced by diffuse traumatic brain injury is linked to disruption of long-range neuronal circuits

**DOI:** 10.1186/s40478-022-01435-3

**Published:** 2022-09-05

**Authors:** Ilknur Özen, Hongcheng Mai, Alessandro De Maio, Karsten Ruscher, Georgios Michalettos, Fredrik Clausen, Michael Gottschalk, Saema Ansar, Sertan Arkan, Ali Erturk, Niklas Marklund

**Affiliations:** 1 Lund Brain Injury Laboratory for Neurosurgical Research, Department of Clinical Sciences, Lund University, Lund, Sweden; 2 Insititute for Tissue Engineering and Regenerative Medicine (iTERM), Helmholtz Zentrum München, Neuherberg, Germany; 3 Institute for Stroke and Dementia Research (ISD), University Hospital, Ludwig Maximilian University of Munich (LMU), Munich, Germany; 4 Section of Neurosurgery, Department of Medical Sciences, Uppsala University, Uppsala, Sweden; 5 Preclinical MRI, Lund University Bioimaging Center, Faculty of Medicine, Lund University, Lund, Sweden; 6 Laboratory for Experimental Brain Research, Department of Clinical Sciences, Lund University, Lund, Sweden; 7 Applied Neurovascular Research for Neurosurgical Research, Department of Clinical Sciences, Lund University, Lund, Sweden; 8 Department of Clinical Sciences Lund, Neurosurgery, Skåne University Hospital, Lund University, Lund, Sweden; 9 Department of Neurosurgery, Skåne University Hospital, 222 20 Lund, Sweden

**Keywords:** Traumatic brain injury (TBI), Demyelination, Axonal injury, Cerebellum, Purkinje cell, Central (midline) fluid percussion, vDISCO

## Abstract

Cerebellar dysfunction is commonly observed following traumatic brain injury (TBI). While direct impact to the cerebellum by TBI is rare, cerebellar pathology may be caused by indirect injury via cortico-cerebellar pathways. To address the hypothesis that degeneration of Purkinje cells (PCs), which constitute the sole output from the cerebellum, is linked to long-range axonal injury and demyelination, we used the central fluid percussion injury (cFPI) model of widespread traumatic axonal injury in mice. Compared to controls, TBI resulted in early PC loss accompanied by alterations in the size of pinceau synapses and levels of non-phosphorylated neurofilament in PCs. A combination of vDISCO tissue clearing technique and immunohistochemistry for vesicular glutamate transporter type 2 show that diffuse TBI decreased mossy and climbing fiber synapses on PCs. At 2 days post-injury, numerous axonal varicosities were found in the cerebellum supported by fractional anisotropy measurements using 9.4 T MRI. The disruption and demyelination of the cortico-cerebellar circuits was associated with poor performance of brain-injured mice in the beam-walk test. Despite a lack of direct input from the injury site to the cerebellum, these findings argue for novel long-range mechanisms causing Purkinje cell injury that likely contribute to cerebellar dysfunction after TBI.

## Introduction

Traumatic brain injury (TBI) is a major cause of morbidity and mortality affecting primarily the young worldwide [1]. It is a heterogeneous disease characterized by primary and secondary injuries [2, 3]. While the primary injury is caused by the initial mechanical impact, the secondary injury is mainly associated with pathophysiological processes that start within hours post-injury and last many years. In TBI, the secondary injury has been reported to cause a range of persistent, long-term symptoms and impairments that include sensory, motor and cognitive deficits [4, 5]. These pathophysiological events are closely associated with the presence of wide-spread axonal injury and white matter atrophy, not only restricted to the more severe cases [5].

The cerebellum has well-recognized roles in balance, coordination, and postural control as well as in the coordination of voluntary movement. In addition, patients with cerebellar lesions often show impaired executive function, distorted spatial cognition, and abnormal behaviour [6]. While the cerebellum may only rarely be directly affected by TBI [7], ataxia, tremor, and motor deficits may be observed after cortical lesions [8, 9], suggesting indirect mechanisms of cerebellar injury. The cerebellum receives the major input from the cerebral cortex via pontine nuclei (PN) that are synaptically intercalated in the cortico-ponto-cerebellar (CPC) pathway. In recent case reports of mild TBI patients, a loss of afferent input from the cortico-cerebellar pathway was attributed to cerebellar dysfunction after mild TBI [10], and atrophy in cerebellar grey and white matter was detected [11, 12]. While indirect cerebellar injury may be a key contributor to the development of post-traumatic morbidity, the underlying pathophysiological mechanisms are not known.

Purkinje cells (PCs) are the sole output of the cerebellum. Therefore, the synaptic cerebellar circuitry depends on strictly regulated PC activity. Neuroanatomical studies using viral approaches in rodents demonstrate region-specific and extensive connectivity between the cerebellum and the cerebral cortex [13]. Plausibly, interruptions of those specific cortico-cerebellar circuits after cortical injury could result in microstructural alterations in PCs. Similarly, reduced number and pathological changes in PCs may affect the output pathways from the cerebellum to the different cortical areas important for cognition and movement. However, it remains unclear to what extent TBI influences PCs, and whether a traumatic cerebral axonal injury mediates a cerebellar dysfunction.

In this study, we combined vDISCO tissue clearing technique that allows unbiased analysis of the whole mouse brain in single-cell resolution and differential tractography based on quantitative anisotropy (QA) to assess alterations in fibers coupled with specific cortico-cerebellar circuits in animals subjected to diffuse TBI using the central fluid percussion injury (cFPI) model in mice. We then performed detailed analyses of PC abnormalities in response to cFPI to assess i) presence of cerebellar axonal swellings, the axonal varicosities; ii) testing of cerebellar function using the beam walk test; iii) structural changes in excitatory and inhibitory synapses of Purkinje cells (PCs); iv) changes in the number of PCs and pinceau size, and v) neurofilament and myelin abnormalities in PCs.

## Material and method

### Animals

Adult male mice C57BL/6 mice (8–12 weeks old; 25-30gr; Taconic, Denmark) and Thy1-GFP-M transgenic mice (8–12 weeks old; 25-30gr; the Jackson Laboratory) were housed with free access to food and water for a minimum of seven days prior to surgery.

### Central fluid percussion model (cFPI) of diffuse traumatic brain injury

The surgical procedure for central fluid percussion injury (C57BL/6 mice cFPI, n = 53; Thy1-GFP-M transgenic mice cFPI, n = 26) has been described in detail previously [14]. Briefly, the mouse was induced in a ventilated Plexiglas chamber with 4% isoflurane in air, and then moved to the stereotaxic frame and anaesthesia was delivered through a nosecone (isoflurane 1.2% and N_2_O/O_2_ 70/30%). Local anaesthesia (bupivacaine, AstraZeneca, Stockholm, Sweden) was injected under the scalp on the top of the head, the skin cut open to reveal the skull. A 3.0 mm diameter craniotomy was made carefully over the midline, keeping the dura mater and the superior sagittal sinus intact. Then, a plastic cap was attached to the skull over the craniotomy using tissue adhesive and secured using dental cement. The cap was filled with isotonic saline at room temperature. The mouse was moved to the fluid percussion device (VCU Biomedical Engineering Facility, Richmond, VA) and connected to the spout of the device. In order to induce a diffuse TBI, the fluid percussion pendulum was released to create a pressure wave subsequently transmitted into the cranial cavity. The injury-induced apnea and immediate post-injury seizures were recorded. Post-injury apnea, observed in all cFPI-injured mice, was 25 ± 15 s (range 10–40 s). Twenty cFPI animals died at time of impact resulting in injury related mortality rate of approximately 17%. Sham-injured animals (C57BL/6 mice Sham, n = 34; Thy1-GFP-M transgenic mice Sham, n = 9) were subjected to anaesthesia and surgery, but the pendulum was not released. The cap was removed following surgery, the bone flap replaced, and the skin sutured using resorbable sutures. The animals were put in a heated cage until they recovered from the anaesthesia and subsequently returned to the home cage.

All animal experiments in the study were randomized and performed blindly by the researchers in areas of the study such as behavior, vDISCO, and MRI at both our and at international research centers. For immunohistochemistry (IHC) analyses, the number of included both C57BL/6 and Thy1-GFP-M mice per group was n = 3 for Sham, and n = 4 for cFPI at all time. For Western blotting analyses the number of included animals (C57BL/6 mice) per group was n = 5 for Sham, n = 8 for cFPI. For vDISCO analyses the number of included Thy1-GFP-M mice per group n = 2 for Sham, n = 11 (total) for cFPI [n = 3 (2dpi), n = 4 (7dpi), n = 4 (30dpi)]. For MRI studies the number of included animals per group was n = 4 for Sham, n = 6 for cFPI. For Beam walking assessment the number of included animals per group was n = 5 for naïve, non-injured animals, n = 5 for Sham, n = 5 for cFPI.

### vDISCO whole-mount immunolabelling of brains

To visualize whole-brain neuronal connectivity, brains of cFPI and sham-injured animals were stained according to the nanobody(V_H_H)-boosted 3D imaging of solvent-cleared organs (vDISCO) whole-mount immunolabeling method [15]. First, the post-fixed brains were incubated in in 4.5 ml of permeabilization solution (1.5% goat serum, 0.5% Triton X-100, 0.5 mM Methylbeta-cyclodextrin (Sigma, 332,615), 0.2% trans-1-Acetyl-4-hydroxy-L-proline (Sigma, 441,562), 0.05% sodium azide (Sigma, 71290) in 0.1 M PBS) for 2 days at 37 °C with gentle shaking. Subsequently, the brains were incubated in 4.5 ml of the permeabilization solution and Atto647N-conjugated anti-GFP nanobooster (Chromotek, gba647n-100) (1:700) for 14 days at 37 °C with gentle shaking. Then, the brains were washed for 2 h 3 times and once overnight with the washing solution (1.5% goat serum, 0.5% Triton X-100, 0.05% of sodium azide in 0.1 M PBS) at room temperature and finally for 2 h 4 times with 0.1 M PBS at room temperature. The immunolabelled brains were cleared with the 3DISCO clearing method [16]. They were first incubated in 4.5 ml of the different gradients of tetrahydrofuran (THF; Sigma, 186,562) diluted in distilled water as follow: 50 Vol% THF, 70 Vol% THF, 80 Vol% THF, 100 Vol% THF and overnight 100 Vol% THF at room temperature with gentle shaking. After dehydration, the samples were incubated for 1 h in dichloromethane, and finally in benzyl alcohol + benzyl benzoate (BABB), (1:2, Sigma, 24122 and W213802) until transparent. During all the clearing steps, the tubes were wrapped with aluminum foil to protect the samples from light.

### Immunofluorescence staining and confocal imaging

Coronal cerebellar Sections (40 μm) were washed three times in PBS, then blocked for one hour in PBS-TX supplemented with 3% NDS (Sigma G9023). Sections were incubated overnight at 4 °C with primary antibodies (see below) diluted in blocking solution. After incubation with primary antibodies, sections were washed three times in PBS-TX then incubated with corresponding secondary antibodies (Life Technologies and Jackson) (1:500 dilution in blocking solution for two hours at RT). Three final washes in PBS were conducted before sections were mounted on slides and images digitally captured using a Zeiss LSM 780 Microscope. Figures were composed using Photoshop CS5 software. Primary antibodies were: MBP (1: 4000, Abcam, Cat No: ab40390), CNPase (1: 500, Cat No: ab6319), SMI-32 (1:1000, Biolegend, Cat No: 801601). Secondary staining was conducted using species-specific fluorophore-conjugated antibodies (Streptavidin Alexa 488, Molecular Probes; Cy3 or Cy5, Jackson).

### Light-sheet microscopy imaging

In the following procedure, whole mouse brain image stacks were acquired using a II ultramicroscope (LaVision BioTec) with an axial resolution of 4 μm and the following filter sets: ex 470/40 nm, ex 640/40 nm. The optic transparent whole mouse brains were acquired individually for High-magnification tile scanning with 4 × objectives (Olympus XLFLUOR 4× corrected/0.28 NA [WD = 10 mm] and PLAN 12x/0.53 NA [WD = 10 mm], LaVision BioTec MI) coupled to an Olympus rotary zoom unit (U-TVCAC) set at 1×. The tile scans were acquired with a 20% overlap and the width of the light-sheet was reduced to achieve maximum illumination in the field of view. The acquired raw images TIFF were processed with Fiji's stitching plugin.

### Western blotting

The cerebellum samples were homogenized by sonication in lysis buffer (20 mM Tris pH 7.5, 150 mM NaCl, 1 mM EDTA, 1 mM EGTA, 1% Triton X-100, 1 mM β-Glycerolphosphate, 1 mM Sodium orthovanadate (Na_3_VO_4_), 1 mM phenylmethylsulfonyl fluoride (PMSF) and Complete™ Protease Inhibitor Cocktail (Sigma-Aldrich) and centrifuged at 14.000 RPM for 20 min at 4 °C. The supernatant was collected and stored at − 80 °C. Protein samples were boiled for 5 min in 2 × Laemelli buffer supplemented with 10% 2-mercaptethanol. Then 10 µg of the samples were subjected to protein separation on Mini-Protean® TGX™ casted gels (Bio-Rad, Hercules, USA); proteins were transferred onto PVDF membranes using a Trans-blot® Turbo™ (Bio-Rad, Hercules, USA) system. Membranes were blocked in TBS (20 mM Tris, 136 mM NaCl, pH 7.6) supplemented with 0.1% Tween 20 and 5% nonfat dry milk before incubation with primary antibodies SMI-32 (1:5000, Biolegend, Cat No. 801601), and Vglut 2 (1:500 Cat No: ab216463) overnight at 4 °C. Signals were boosted by binding of horseradish peroxidase (HRP)-linked secondary antibodies (anti rabbit 1:25,000 and anti-mouse 1:10,000, Sigma-Aldrich, Cat No: A0545) to the primary antibodies. Membranes were stripped and reprobed for β-actin (1:75,000, Sigma-Aldrich, Cat No: A3854). Membranes were exposed on a ChemiDoc™ MP system (BioRad) using a chemiluminescence kit (Merck Millipore, Billerica, MA, USA) that reacted with the HRP-linked secondary antibodies. Densitometry analyses were conducted using ImageJ software (Billerica, MA, USA) and protein levels were calculated as percentage of β-actin expression.

### MRI experiments

Data were acquired on a 9.4 T Agilent magnet (Agilent, Santa Clara, USA) equipped with Bruker BioSpec AVIII electronics operating with 7.0.0 (PV7) a BGA 12S HP gradient system (Bruker, Ettlingen, Germany) with a maximum gradient strength of 670 mT/m and a rise time of 130 µs. The measurements were performed with a transmit-receive quadrature cryo coil from Bruker.

#### Anatomical imaging

High-resolution T2-weighted images were obtained with a 2D RARE sequence. The following parameters were used: TE 44 ms, RARE factor 10, TR 3.4 s, resolution 50 × 50 mm^2^, FOV 18 × 15 mm^2^ and slice thickness 0.5 mm. 32 slices were acquired axially with 6 averages in 10 m 12 s.

#### Diffusion tensor imaging

Diffusion tensor imaging was performed with 2D EPI readout with TE 20 ms, TR 6 s and a spectral bandwidth of 250 kHz. An isotropic resolution was used with in plane resolution of 170 × 170 um^2^, FOV 11 × 8 mm^2^ and slice thickness 0.17 mm with 80 slices. 64 diffusion directions were used with 5 reference images with no diffusion gradient. The B value was 2500 mm^2^ s^−1^ and the total acquisition time 34 m 30 s with 5 averages.

#### Fiber Tractography

Diffusion data preprocessing involved four steps: manual brain extraction, head motion and eddy currents correction via the eddy method provided by FSL [17], images registration and group template construction. The b-zero volumes (b = 0 s/mm2) of each imaging session were longitudinally registered using the ANTs rigid scheme then a template for each temporal (1 and 7 dpi) and experimental (TBI, sham) condition was constructed [18]. Generalized q-sampling imaging [19] (GQI) was used to calculate the orientational distribution of the density of water diffusion, then quantitative anisotropy (QA) estimated for each within-voxel fiber orientation. Template transforms were then applied to the QA metric maps and longitudinally compared using differential tractography [20] (threshold difference ≥ 30%). Seeding regions were separately placed at the cerebellum and whole brain, while the number of tracts resulting from decreased QA were analysed via false discovery rate (FDR).

### Motor coordination assessment

The beam walk test was performed as previously described [21]. The mouse was placed on a wooden beam (5 cm × 4 mm and 80 cm length) with the home cage placed at the end of the beam. The mouse traversed the beam to reach the home cage. Before being subjected to TBI or sham injury the mice were trained to cross the beam until a stable baseline performance was obtained. The performance of mice was recorded with a video camera, and the total number of steps and faults for each paw counted by a trained and blinded observer. The beam walk test was performed between 9 and 11 am.

### Statistics

Graphs and statistical analysis were made with GraphPad Prism 8 (GraphPad Software, La Jolla, CA, USA). Kruskal–Wallis one-way ANOVA with Dunn’s post-test was used following analyses of normality of data distribution by using Shapiro–Wilk normality test. Statistical analyses for Western blot results were performed with Student’s t test between sham-injured and cFPI group for each time point. All data are expressed as the mean ± S.E.M. Significance was set at *P* < 0.05.

## Results

### Disruption of mossy fiber synapses in the cerebellum after diffuse traumatic brain injury

**Fig. 1:**
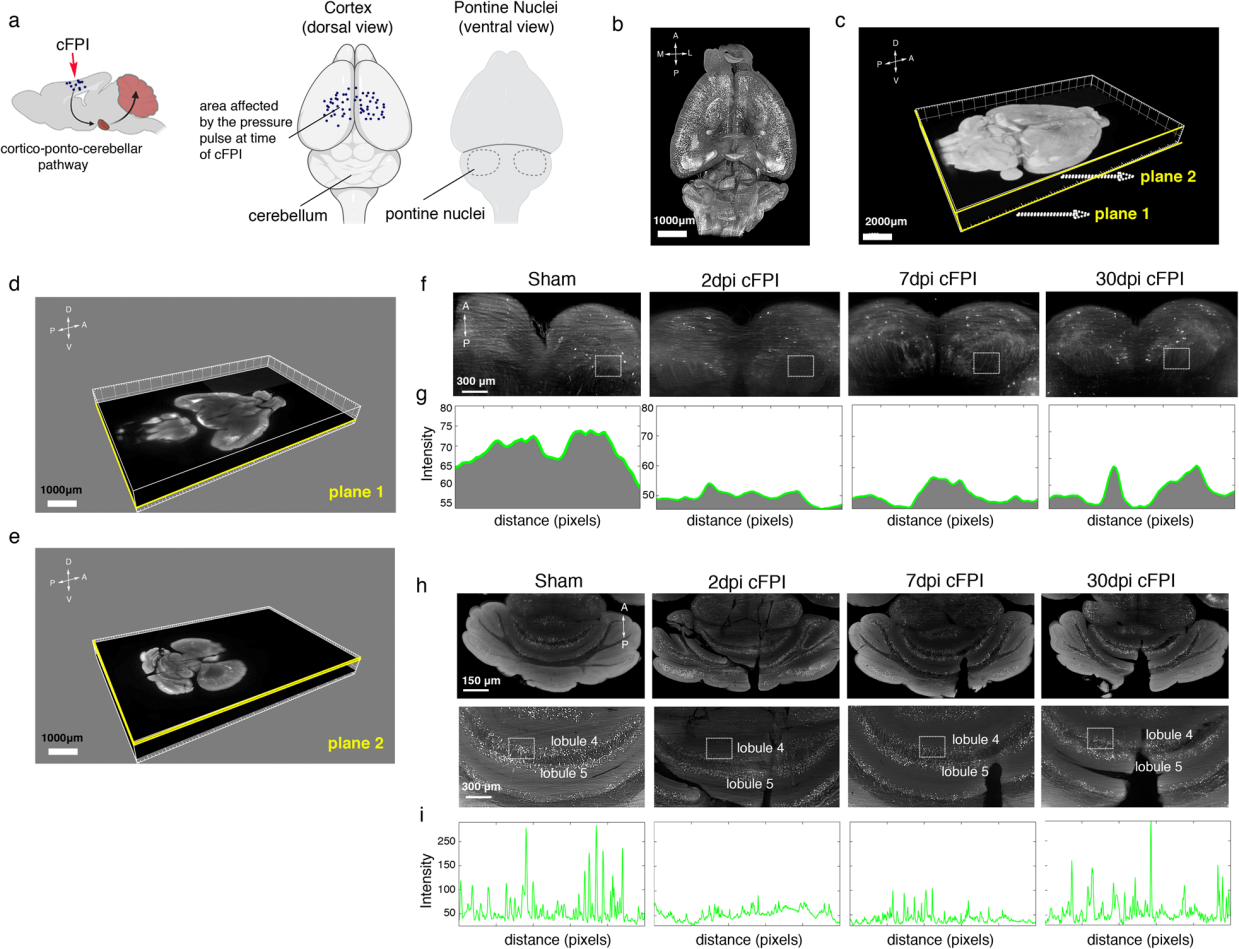
Traumatic brain injury causes early changes in mossy fiber terminals in the cerebellum. **a** Schematic diagram of the cortico-ponto-cerebellar circuit in a Thy1-GFP-M mouse mouse subjected to cFPI. Illustrations of mouse brains showing the area impacted by the pressure pulse at time of cFPI, the cerebellum (dorsal view) and the pontine nuclei (ventral view). **b** Light-sheet microscopy imaging of the cerebral hemispheres and cerebellum cleared with vDISCO shows highly detailed neuronal structure and axonal projections. **c–e** 3D reconstruction of cleared whole brain of a Thy1-GFP-M mouse. **c** Two selected representative horizontal planes at **d** pontine nuclei (plane 1) and **e** cerebellum (plane 2) are shown. **f** High-magnification representative fluorescence images of the pontine nuclei of a sham-injured mouse and brain-injured mice at 2, 7 and 30 dpi. **g)** Plots of signal intensity profiles from boxed areas in **f** in the pontine nuclei of a sham-injured mouse, and brain-injured mice at 2, 7 and 30 dpi. **h** High-magnification, representative fluorescence images of the vermis lobules IV/V of the cerebellum of a sham-injured mouse and brain-injured mice at 2, 7 and 30 dpi. **i** Plots of signal intensity profiles from the boxed areas in **h** in the vermis lobule IV/V of a sham-injured mouse and brain-injured mice cerebellum at 2, 7 and 30 dpi

The cerebellum receives the major input from the cerebral cortex via pontine nuclei (PN). Mossy fiber (MF) axons, mainly glutamatergic, projecting from PN to multiple cerebellar lobules innervate the granule layer by forming large bouton-like terminals named rosettes. Although accumulating evidence has revealed a complex interaction between the cerebral cortex and different cerebellar lobules through the cortico-ponto-cerebellar pathways [13], anatomical changes in mossy fibers innervation to cerebellum after TBI have not been described. Therefore, we first used the tissue clearing vDISCO method, an organic-solvent-based-method that allows for whole-brain imaging with high transparency [15]. Using this technique in combination with a light-sheet microscope, we examined the effects of diffuse TBI on the axons of pontine nuclei and MF terminals in the granular layer of the cerebellum of Thy1-GFP-M mice (Fig. 1). Horizontal planes at different depths were extracted from the reconstructed 3D mouse brain to examine axonal and neuronal changes in the pontine nuclei and cerebellum of injured Thy1-GFP-M mice (Fig. 1a–g). The intensity of GFP + axons in the pontine nuclei of injured Thy1-GFP-M mice at 2, 7 and 30 dpi was lower than sham control (Fig. 1f–g). Similarly, we observed that intensity of MF axon terminals in the granule layer of lobule 4/5 was decreased in the brain-injured mice at 2, 7 and 30 dpi when compared to sham controls (Fig. 1h, i).

### Rapid formation of axonal varicosities in the injured cerebellum and motor deficits in brain-injured animals

**Fig. 2:**
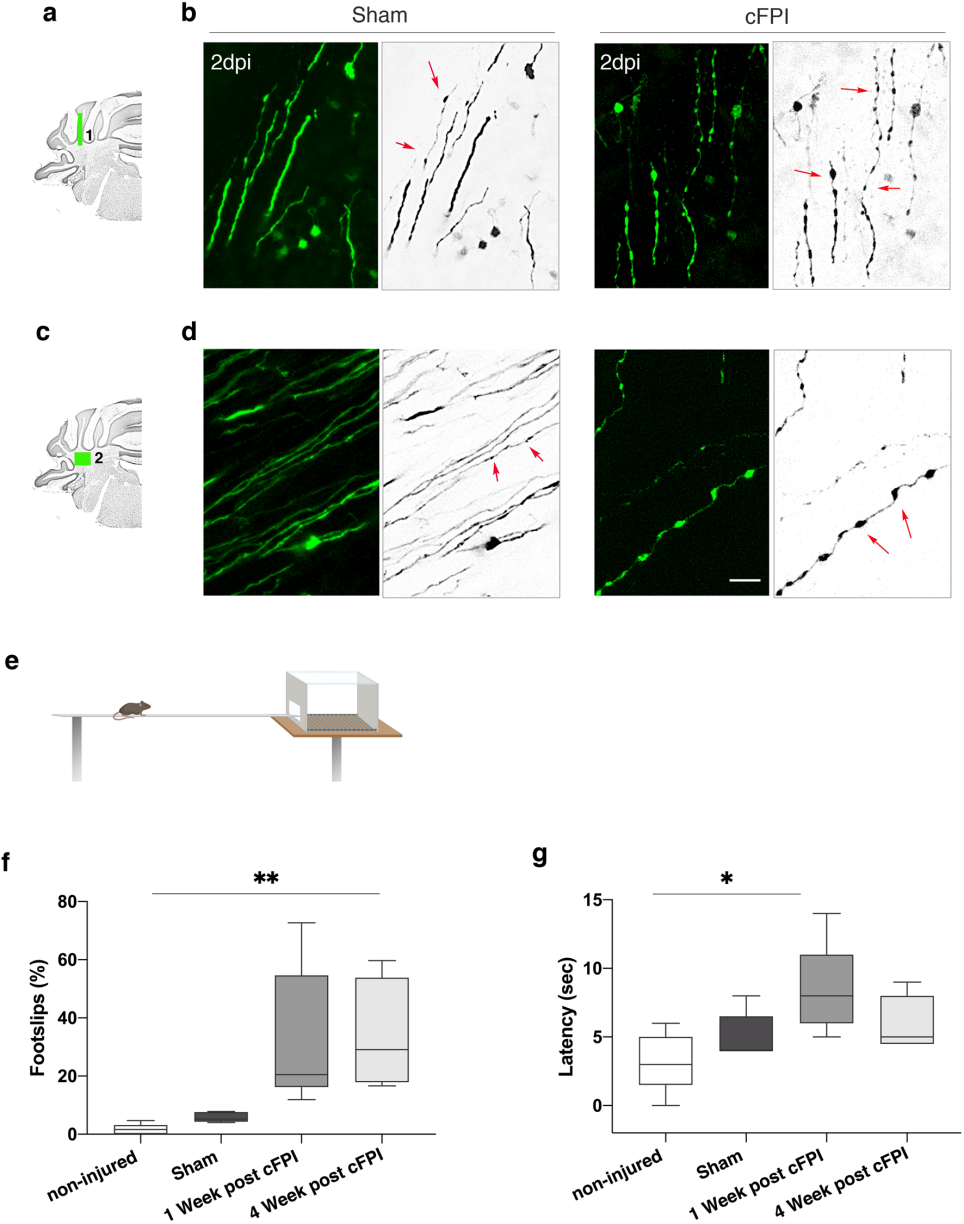
Traumatic brain injury causes early formation of axonal varicosities in the cerebellar white matter and impairment of motor function. **a,c)** Schematic diagram of spatial distribution of axonal varicosities in the different regions of cerebellar white matter. (1) In long-range pathways that terminates as mossy fibers and (2) in the region of cerebellar nuclei (2) **b** Confocal images of varicosities along GFP + axons (green) axons in green boxed area **(1)** in the cFPI injured Thy1-GFP-M mice. **d** Confocal images of varicosities along GFP + axons (green) axons in green-boxed area **(2)** in the cFPI injured mice. There were only few or no varicosities in the sham-injured mice in the regions marked by the green boxes in **a** and **c**. GFP signals are inverted into grey scale. Red arrows point to axonal varicosities. Scale bar 20 μm. **e** Schematic diagram of mice while performing the beam walk test. **f** Reduced limb coordination of cFPI mice in the beam walk test. Percentage of hindlimb slips in the non-injured, sham and cFPI mice at 1 week and 4 weeks after the injury. **g** The average time to cross the beam significantly decreases in the cFPI at 7 days after the injury (**P < 0.001; (*P < 0.01, n = 5 (non-injured, naïve), n = 5 (Sham), n = 5 (cFPI), Kruskal–Wallis followed by Dunn’s post hoc test)

Purkinje cells dysfunction may be associated with axonal swelling during early stage of the injury that precedes demyelination in the cerebellum white matter [7]. To test this hypothesis, we performed confocal microscopy on Thy1-GFP-M mice (Fig. 2a–d). GFP signalling was clear and allowed to visualize morphological changes in axons of cerebellar white matter of brain-injured mice. At two days after the TBI, axonal varicosities were observed in the cerebellar white matter of Thy1-GFP-M transgenic mice (Fig. 2a–d) despite the lack of direct input from the cortex to the cerebellum. Then behavioural assessment was performed using the beam walk test. The cFPI mice showed impairment in beam-walking ability causing increased percentage of foot slips, compared to the non-injured animals at weeks 1 and 4 (Fig. 2e,f). These impairments in cFPI mice were the most evident at 1 week after the injury, resulting in longer beam latencies. However, this was not significantly different in the cFPI mice at week 4 compared with sham-injured animals (Fig. 2g). There were no significant differences with regard to foot slips and beam latencies between naïve, non-injured animals and sham-injured animals (Fig. 2f, g).

### Changes in excitatory and inhibitory synapses in Purkinje cells following diffuse traumatic brain injury

**Fig. 3:**
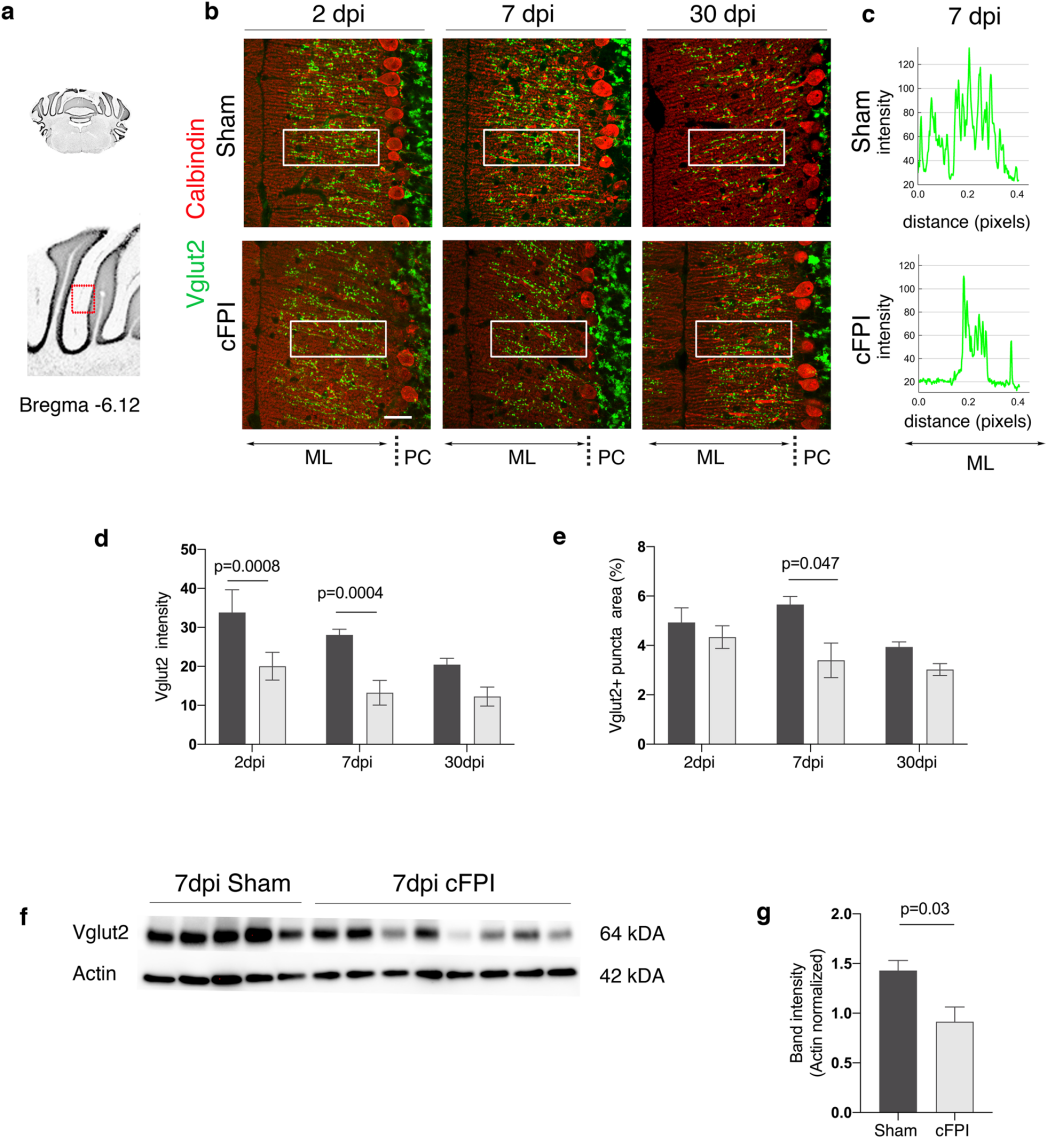
Decreased Vglut2 expression in the cerebellum of brain-injured mice at seven days after TBI. **a** Schematic of anterior coronal cerebellum sections (bregma -6.12). **b** Immunofluorescence labelling for Vglut2 (green) and calbindin (red) in the ML of the cerebellum of sham-injured and cFPI mice at 2, 7, and 30 dpi. **c)** Plots of signal fluorescence intensity profiles of Vglut2 expression from boxed areas in the cerebellar molecular layer (ML) of sham-injured and cFPI mice at 7dpi. **d**, **e** Quantification of Vglut2 density (**d**) and numbers of Vglut2 + puncta (**e**) from boxed areas in the ML at all time points after the injury. **f**, **g** Representative Western blots (**f**) and quantification (**f**, **g**) of Vglut2 band intensity in sham-injured and cFPI mice at 7 dpi (mean ± SEM; n = 5 (Sham), n = 8 (cFPI); two-tailed Student’s *t-*test, unpaired). *PC* Purkinje cells, *dpi* days post-injury, *cFPI* central fluid percussion injury, *Vglut2* vesicular glutamate transporter-2

Climbing fiber (CF) axons, the second major input to the cerebellum, project from the inferior olivary nuclei to innervate PCs that modulate motor output. They form synapses on PC dendrites and specifically express Vglut2 [22, 23]. We hypothesized that diffuse TBI can cause changes in the innervation of CFs onto Purkinje dendrites in the molecular layer. To test this, we performed double immunofluorescence staining for Vglut2 and calbindin_D28K_ (Fig. 3). Vglut2 expression was found as a punctate pattern in the molecular layer as well as the glomeruli in the granule layer (Fig. 3a, b). We then analysed fluorescence intensity of Vglut2 in the molecular layer, and observed a significant decrease in brain-injured mice when compared to controls at 2 and 7 dpi (Fig. 3c, d). The percentage of Vglut2 puncta on molecular layer was lower in the brain-injured mice when compared to sham-injured animals, but was not significantly different from sham at 2 and 30 dpi (Fig. 3e). Consistently, Western blot analyses showed that Vglut2 expression in the cerebellum of cFPI mice was significantly decreased at 7 dpi (Fig. 3f, g).

### Diffuse traumatic brain injury causes abnormal neurofilament expression and reduced pinceau size in Purkinje cells

**Fig. 4:**
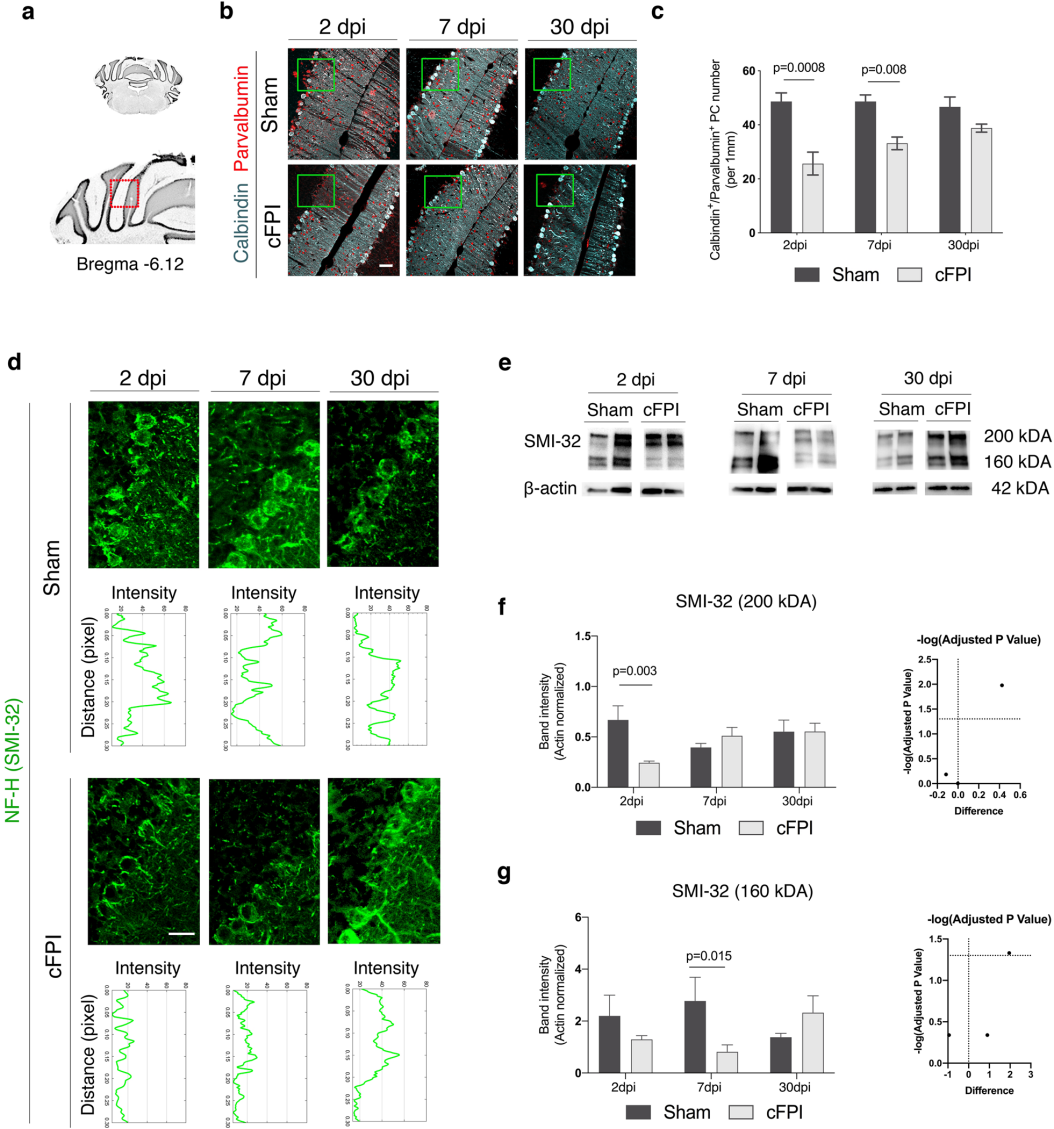
Reduced calbindin, parvalbumin, and non-phosphorylated neurofilament expression in the Purkinje cells after traumatic brain injury. **a** Schematic of anterior coronal cerebellum sections (bregma-6.12). **b**, **c** Representative confocal images (**b**) and quantification (**c**) of Purkinje cells (PCs) in the red boxed area in **a** co-labelled for calbindin (cyan) and parvalbumin (red**)** in sham-injured and cFPI mice at 2, 7, and 30 dpi**,** scale 50 μm. **d** High magnification confocal images and fluorescence intensity profiles of non-phosphorylated neurofilament-H (SMI-32) expression in Purkinje cells in the sham-injured and brain-injured mice at 2, 7 and 30 dpi in green boxed areas in **b**, scale 20 μm. **e–g** Representative Western blots (**e**) and quantification (**f, g**) of SMI-32 (200kDA) and SMI-32 (160kDA) band intensity in sham-injured and cFPI mice at 2, 7 and 30 dpi (mean ± SEM; n = 5 (Sham), n = 8 (cFPI); two-tailed Student’s *t-*test, unpaired). *dpi* days post-injury

To investigate whether forebrain-targeted cFPI causes PC loss, we performed immunohistochemistry on cerebellar sections for calbindin_D28K_ and parvalbumin (PV) (Fig. 4a–c). Expression of both calcium-binding proteins was observed in PC bodies in sham and cFPI mice. Purkinje cells were found between molecular and granular layers, and had clearly defined round cell bodies (Fig. 4b). The numbers of PCs were significantly lower in cFPI mice than in sham-injured animals at 2 dpi and 7 dpi (Fig. 4b, c).

Neurofilaments provide mechanical stability to neurons and their axons. In order to further understand TBI-induced pathological changes in PCs, we analysed non-phosphorylated neurofilaments (NFH) SMI-32 expression in PCs. Under physiological conditions, SMI-32 expression is mainly found in PC bodies. Analyses for fluorescence intensity of SMI-32 signalling in PCs cell layer showed a significant decrease at 2dpi and 7dpi in cFPI animals compared to sham-injured mice, but not at 30dpi (Fig. 4d). We then performed Western blot analysis using a non-NFH SMI-32 antibody that recognizes the heavy chain of a protein of 200 kDA; however lower molecular weights products were also observed in the injured animals (Fig. 4e). Densitometry for SMI-32 (200 kDA) showed a significant decrease at 2dpi in cerebellar lysates derived from cFPI animals compared to sham-injured mice, but not at 7 and 30dpi (Fig. 4f). In contrast, there was a significant decrease in the intensity of lower molecular weight products (160 kDA) in cFPI animals when compared to sham-injured mice at 7-day post injury (Fig. 4g).

**Fig. 5:**
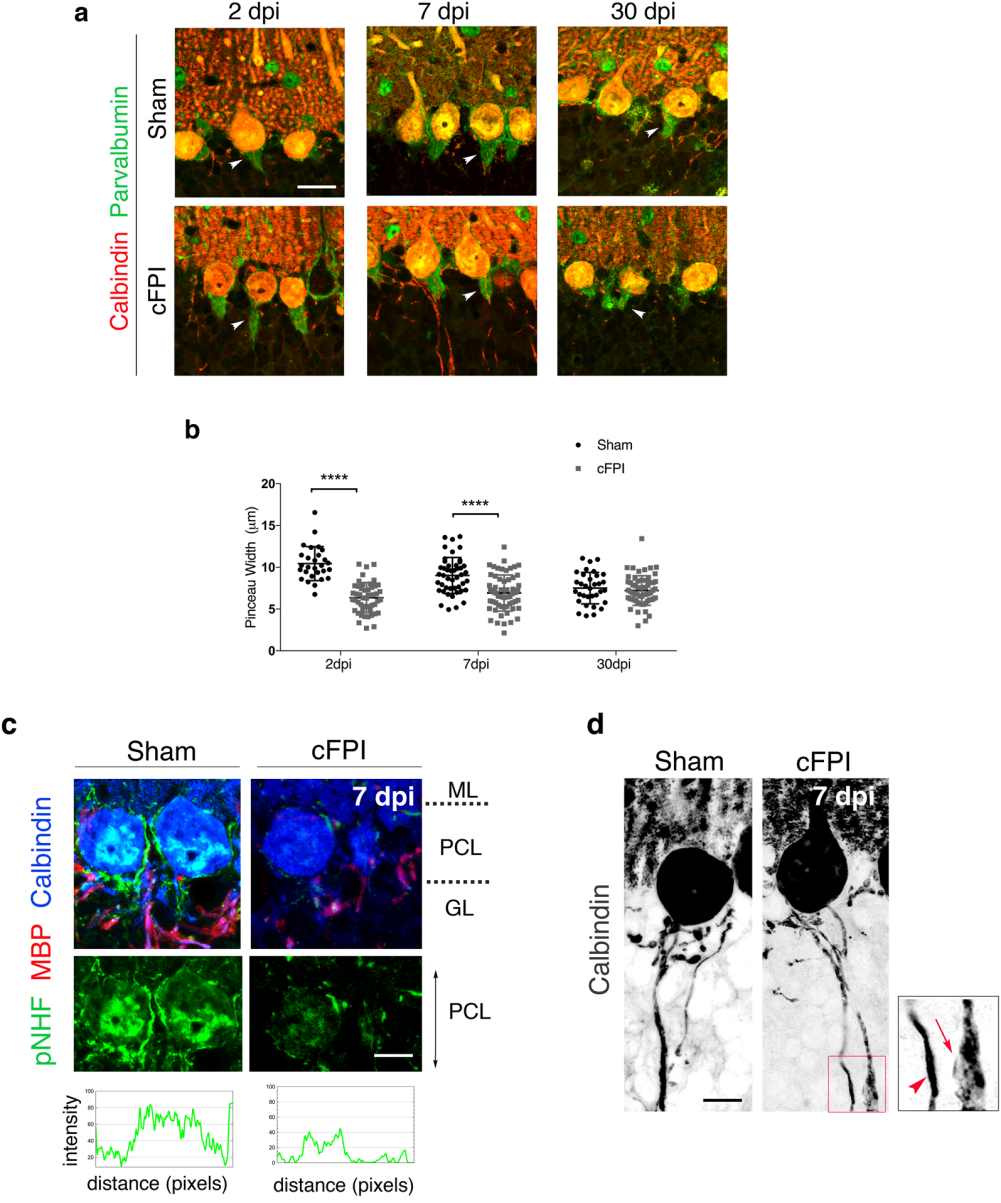
Decreased pinceau synapse size and phosphorylated neurofilament in Purkinje cells is associated with axonal swellings after traumatic brain injury. **a**, **b** Disrupted pinceau synapses in the cerebellum following cFPI. **a** Confocal images showing co- labelling of calbindin (red) and parvalbumin (green) in the cerebellum of sham and cFPI mice at 2, 7 and 30 dpi, scale bar 10 μm. White arrowheads indicate pinceau width. **b** Quantification of pinceau width (μm) in the cerebellum of sham and cFPI mice at 2, 7 and 30 dpi (****P < 0.0001; Kruskal–Wallis followed by Dunn’s post hoc test). **c** Confocal images showing triple labelling of pNF-H (green), MBP (red), and calbindin (grey) of Purkinje cells in sham and cFPI mice at 7dpi. Fluorescence intensity profiles of pNF-H (green) expression in the PCL of sham-injured and cFPI mice cerebellum at 7dpi. **d** Morphological changes in calbindin-expressing Purkinje cells axons, including axonal swellings and thickening, in the brain- injured animals as compared to sham-injured controls at 7dpi. Confocal pictures are inverted into grey scale. Scale bars 10 μm in **c** and **d**. ML: molecular layer, PCL: Purkinje cell layer, GL: granular layer. pNF-H: phosphorylated neurofilament-heavy, MBP: myelin basic protein

The pinceau, cone-shaped structures at the base of PCs, are perisomatic synapses formed by basket cells that send inhibitory input to PCs at the axon initial segment (Fig. 5). They are shown to regulate initiation of spike output from PCs [24]. Alterations in pinceau formations have been described as morphological features of PC dysfunction in the degenerating cerebellum [25]. Therefore, we next analyzed parvalbumin (PV) expressing pinceau plexuses that formed a distinct cone shape surrounding the PC axon initial segment (Fig. 5a). The percentage of PCs that contained PV^+^ plexus in the injured mice remained unchanged compared to sham-injured mice at all time points. However, there was a significant reduction in the size of PV^+^ pinceau synapse width (Fig. 5b).

### Loss of myelin in Purkinje cells and cortico-cerebellar white matter tracts

**Fig. 6:**
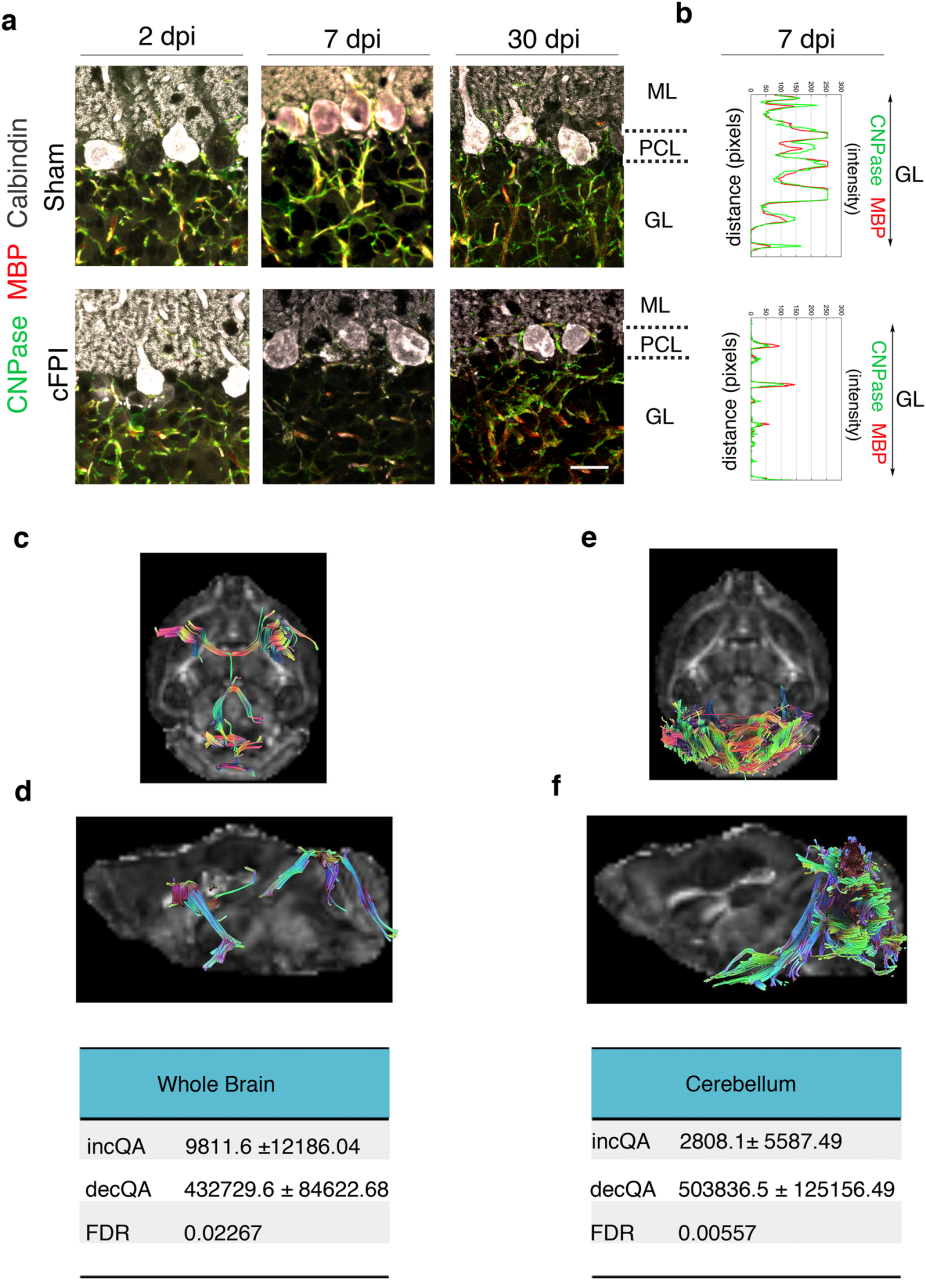
Reduced myelin proteins and quantitative anisotropy in the mouse brain following TBI. **a** Confocal images showing triple labelling of CNPase (green), MBP (red), and calbindin (grey) of Purkinje cells of sham and cFPI mice at 2, 7 and 30 dpi, scale bar 10 μm. **b** Fluorescence intensity profiles of MBP (red) and CNPase (green) expression in the granular layer (GL) of sham-injured and cFPI mice cerebellum at 7dpi. **c–f** Representative fiber tracts with decreased QA in the whole brain (**c**) and cerebellum (**f**) of injured mice longitudinally traced from one day to seven days after the injury. **c, d** In the whole brain, obtained from an average number of tracts with increased QA signal 9811.6 ± 12,186.04 and an average number of tracts with decreased QA signal 432,729.6 ± 84,622.68, FDR for tracts visualized 0.002; **e**, **f** In the cerebellum, obtained from an average number of tracts with increased QA signal 2808 ± 15,587.49 and an average number of tracts with decreased QA signal 503,836.5 ± 125,156.49, FDR 0.005. *QA* Quantitative anisotropy, *FDR* False discovery rate, *ML* molecular layer; *PCL* Purkinje cell layer, *GL* granular layer, *dpi* days post-injury, *CNpase* 2′,3′-cyclic nucleotide 3′-phosphodiesterase, *MBP* myelin basic protein

Our previous study demonstrated that reduced phosphorylated neurofilament (pNHF) protein levels are correlated with decreased myelin levels in the cerebellum [7]. To investigate if pathological changes in PCs may be due to myelin loss and reduced pNHF after diffuse TBI, we performed immunohistochemistry on cerebellar sections for MBP and pNHF (SMI-31) (Fig. 5c). Analyses for fluorescence intensity of SMI-31 signalling in PCs cell layer showed a significant decrease in PCs at 7dpi in cFPI animals compared to sham mice (Fig. 5d). Immunofluorescence analyses further revealed that both CNPase and MBP expression was significantly decreased in the Purkinje cells and their axons in the granular layer at 7dpi (Fig. 6a, b).

When seeding in the whole mouse brain, longitudinal differential tractography obtained an average number of tracts with increased QA signal of 9811.6 ± 12,186.0, while 432,729.6 ± 84,622.7 with decreased QA, resulting in an FDR 0.002. Similarly, when seeding in the cerebellum, the average number of tracts found with increased and decreased QA were 2808 ± 15,587.5 and 503,836.5 ± 125,156.5, respectively; FDR 0.005 (Fig. 6c-f).

## Discussion

The cerebellum is an integral part of motor function and coordination, but also of higher-order cortical functions. Using the central fluid percussion injury (cFPI), a TBI model that causes widespread axonal injury in mice, we observed numerous cerebellar alterations although the cerebellum is not directly affected by the impact delivered to the cerebral cortex. We found that microstructural alterations of PCs were related to the disruption of specific fibers of the cortico-cerebellar circuits along with demyelination. The Purkinje cell (PC) pathology may be related to axonal degeneration, including axonal varicosities, observed at long-range following TBI. The mechanical forces affecting the brain at time of TBI cause alterations in brain function due to e.g. disruptive processes in the neuronal circuits and the white matter. In response to trauma, these mechanical forces may affect distinct regions in the brain remote from the site of the initial head impacted [7, 26–28]. The degree of force influencing the cerebellum in our mouse fluid percussion model is not known, while previous work in the lateral fluid percussion model in rats found that the injury resulted in a degree of strain in the cerebellum, albeit at a lower level than other brain regions[30]. While the biomechanics are different from those of our model, blast injuries result in cerebellar pathology both in the mouse as well as in the human setting [31]. In contrast, athletes with persistent post-concussive symptoms suffering from vestibular impairment were recently observed to display merely minimal changes in cerebellar white matter [29]. Thus, the type of injury may result in different degree of cerebellar pathology. Clinically, cerebellar dysfunction induced by TBI commonly results in neurological symptoms and is of relevance to veterans, athletes, and survivors of other TBI subtypes. Given that the pathology of diffuse TBI is closely associated with the mechanical forces of the primary injury, it is important to understand the interplay between the mechanics of the forces applied to the forebrain and pathological changes in the cerebellum not primarily affected by the injury.

Diffuse axonal injury is a clinical entity often observed following closed head injury. Clinical and experimental studies show selective vulnerability of white matter axons to the mechanical impact that occurs as a result of rapid head linear accelerations [32]. Neural circuit dysfunction caused by injury to neurons, axons and synapse formation following TBI can impair neural information processing. Studies using viral tracing in rodents demonstrate region specific and extensive two or three-synaptic connectivity between the cerebellum and the cortex, highly associated with human brain anatomy [13, 33]. The CPC connects the cerebellum with different cortical areas involved in motor coordination and movement [13]. Mossy fibers, the largest component of the CPC pathway, project from pontine nuclei to the granular layer of cerebellar cortex that contains excitatory granule cells. Here, we observed, for the first time, the vulnerability of afferent/efferent inputs from the cortico-cerebellar pathway and PC pathology following indirect brain injury. By using vDISCO, we first constructed the whole brain neuronal connectivity map of Thy1-GFP-M transgenic mouse subjected to diffuse TBI [15]. When we visualized the details of neuronal connections in the pontine nucleus and innervating mossy fiber (MF) terminals to injured cerebellum, there was a rapid decrease in the number of GFP-labelled MFs in lobules IV/V of the cerebellum at the early time points (2 and 7 days). Lobules IV/V receive mainly motor input from the ponto-cerebellar pathway as well as higher cortical areas [13, 34]. The beam walk test showed that diffuse TBI caused significant impairments of motor coordination assessed using the beam walk test. Our previous studies showed that cFPI results in complex behavioural disturbances [35]. Similar to studies using the controlled impact model, we showed that mice subjected to cFPI had more foot slips on the beam and longer traversing latency starting from 1 to 4 weeks post-injury [36]. In contrast, the latency to cross the balance beam was increased at 1 week but there were no significant difference when compared to controls (sham and non-injured) at 4 weeks. This suggests that difference between observed motor defects and latency could be a manifestation of different cerebellar circuits being recruited due to topographical organization of the cerebellar cortex at different time points after the injury. In terms of the complexity of cortico-ponto-cerebellar pathways, one possibility could be the involvement of other brain regions, such as pontine nuclei and basal ganglia converging on different subset of Purkinje cells with altered synaptic input and connectivity patterns in the damaged cerebellum. On the other hand, cFPI also results in striatal pathology, in particular in the globus pallidus, which may also have influenced the beam walk test [37]. However, while not specific for cerebellar function this test remains sensitive in the assessment of cerebellar pathology [28].

Under the pathological conditions, alterations in climbing fiber synapses in the molecular layer can occur due to PC degeneration and/or persistent abnormal activity of climbing fibers [38, 39]. For instance, abnormal climbing fiber PC connections including decreased VGlut2 synaptic density was reported in the post-mortem cerebellar tissue of essential tremor cases [38]. Therefore, it is likely that the modulation of climbing fibers and their synapses on PCs in the molecular layer of TBI mice can be dynamic, as observed in degenerative disorders associated with cerebellum pathology [38, 40]. We found a significant decrease in VGlut2 synaptic density in the molecular layer of the cerebellum of cFPI-injured mice at 7 days. This was correlated with reduced protein levels of Vglut2 in the cerebellar tissue lysates from 7 days post-injury. VGlut2 is one of the major pathway of excitatory neurotransmission in the cerebellum [41]. The glutamate–mediated excitotoxicity is a rapid event that can lead to neurodegeneration, and is a well-established injury mechanism in experimental TBI models. For instance, a presynaptic hyperexcitation was observed following TBI in rats from day 3 to day 7 post- injury [42]. Therefore, our data support the hypothesis that alterations in mossy and climbing fibers could be a compensatory mechanism as a result of a TBI-induced glutamate–mediated pre-synaptic hyperexcitability in the Purkinje cells. However, further functional studies are needed to understand the underlying mechanisms in TBI.

Neurofilaments (NFs) are integral components of the axonal cytoskeleton and shown to be involved in structural plasticity in GABAegic and glutamatergic synapses in different neurodegenerative disorders [43, 44]. Since SMI-32 is an established marker for axonal damage[45], NFs may also have a role in PCs degeneration after TBI. We found a significant decrease in the expression of non-phosphorylated NF (SMI-32) in the PC layer of cFPI mice. Similarly, there was a decrease in phosphorylated (pNF-H) NF immunoreactivity in the PC layer, and we observed low molecular weight bands of SMI-32 that may indicate breakdown products by calpain activity [46]. Consistently, we have recently reported that decreased SMI-31 immunoreactivity in the cerebellum was mainly observed in the areas of myelin loss after diffuse TBI [7].

Purkinje cell loss has been reported in rodent studies using closed cortical impact and fluid percussion injury models of TBI [26, 47, 48]. Although contributing mechanisms were not explored, mild fluid percussion injury in rats has previously been shown to induce a significant loss in the number of Purkinje cells in the cerebellar vermis where activated microglia cells were found in close proximity to the bodies and dendritic arborisation of Purkinje cells. These distinctive patterns of Purkinje cell loss and microglia activation vary in accordance with injury severity and were detected starting as early as 3 h and persisting up to 7 days post-injury [26, 49]. Consistently, we found a significant reduction in the number of calbindin_D28k_-expressing PCs at 2 and 7 days post- injury [47]. The calbindin_D28k_ immunostaining also revealed PCs axonal swellings, named torpedoes[50], which may be early subcellular event induced by PC degeneration. We also observed, at long-range from the cortical impact, widespread axonal varicosities in the cerebellum. Axonal varicosities are axonal swellings, important hallmarks of traumatic axonal injury that may indicate disruptions in the myelin sheath or axonal cytoskeleton causing disrupted axonal transport. Using Thy1-GFP-M transgenic mice, we could visualize axonal varicosity formation in the cerebellum white matter after the injury. Several studies using either closed skull or fluid percussion TBI rodent models reported axonal varicosities in the corpus callosum shortly after the single mechanical impact to the cortex of Thy1-YFP transgenic mice [51, 52]. Our data shows, for the first time, that axonal varicosities could be also induced in remote regions following cFPI.

In Purkinje cells, progressive myelin loss was associated with extensive axonal changes including axonal swellings and neurofilament dephosphorylation in rat model of chronic, nonimmune-mediated demyelination [53]. We previously showed that the cFPI model results in progressive myelin loss and axon-myelin disruption in the cerebellum [7]. Cytosketal disruption can lead to axonal swelling and ultimately to axonal transections resulting in loss of synaptic function. Our data argue for trans-synaptic injury mechanisms occurring in remote regions after the initial mechanical injury distributed to the cortex [54]. However, detailed analyses of specific cortico-cerebellar circuits have not previously been performed. Here, significant abnormalities in the myelinated projection fibers carrying information to and/or from the cerebellum were found. Reduced myelination and decreased fiber density are associated with lower levels of quantitative anisotropy (QA). The lower QA measurements in the cFPI compared to sham controls represented longitudinal injury evolution between post-injury day 1 and day 7 involving the major white matter tracts seeding in the corpus callosum and cerebellum.

Understanding the key pathologic changes in the cerebellum, caused by long-range axonal pathology, may help design therapies aimed at improving clinical outcome of TBI. Our findings identify that structural changes in Purkinje cells induced by indirect injury is closely related to motor deficits. However, this study is not without its limitations. Although our results show that cortical injury causes significant structural changes in the synapses of PCs, electrophysiological studies are needed to explore firing rates and hyperexcitability that may occur in PCs. Tract-tracing studies could reveal mechanisms for transsynaptic transmission of factors causing long-range changes in the cerebellum. Other assessments of higher-order and cognitive functions have not been tested in the study, while such impairments are well-established in this model of diffuse TBI in mice [55]. Finally, TBI-induced Purkinje cell dysfunctions in humans remain to be investigated.

## Data Availability

All data generated or analysed in this manuscript are included in the article. All requests for data may be sent to the corresponding author.
